# Cattle recover completely from unconsciousness induced by controlled application of 150–180 kJ of 915 MHz microwave energy to the forehead

**DOI:** 10.1016/j.vas.2025.100466

**Published:** 2025-06-08

**Authors:** Alison Small, Ian Jenson, Andrew Phillips, David McLean, Troy Kalinowski, James Ralph

**Affiliations:** a FD McMaster Laboratory, Agriculture and Food, CSIRO, Locked Bag 1, Armidale NSW 2350, Australia; b FIRST Management Pty Ltd, Barramattigal Country, PO Box 2523, North Parramatta NSW 1750, Australia; c Wagstaff Food Services Pty Ltd, 1500 Thompson Road, Cranbourne VIC 3977 Australia; d Advanced Microwave Technologies, 34 Dobbie Avenue, East Corimal NSW 2518, Australia

**Keywords:** Humane slaughter, Beef, Welfare, Religious slaughter, Diathermic syncope, Electromagnetic, Stunning

## Abstract

For beef to be acceptable to consumers of Halal and Kosher meat, the animal must be alive at the point of exsanguination, undamaged and, if rendered unconscious prior to slaughter, able to recover from the unconscious state if not exsanguinated. A dielectric (electromagnetic) system, trademarked DTS: Diathermic Syncope® (DTS) has the potential to address these requirements. Seven cattle were rendered unconscious using DTS at 150–180 kJ of energy. Electroencephalography (EEG) was performed prior to and after DTS energy application in 6 of these animals, while they recovered consciousness, while the seventh was handled in accordance with commercial processing procedures, without the delays involved in collecting EEG data. Five animals (4 of which had EEG recorded) were returned to the holding pen after recovery and held for a period of 30 min, during which they explored their surroundings ate hay and rested. After the observation period, they were returned to restraint, DTS energy reapplied and the carcases processed for human consumption. Loss of consciousness, based on behavioural reactions, occurred within 10 s of the start of energy application, while EEG indicated the duration of insensibility to be 63 s (based on absence of eye reflexes) or more, with a long transition to full return of consciousness. Animals recovered fully from the induced unconsciousness and responded normally to environmental stimuli. No evidence of aversion was noted when these animals were returned to the restraint box a second time, some 30 min after recovery.

## Highlights

• Seven cattle were rendered unconscious, then recovered.
• Behavioural signs of unconsciousness occurred within 10 s of the start of energy application.
• EEG indicated that the duration of unconsciousness was at least 63 s.
• Animals recovered fully from unconsciousness and responded normally to environmental stimuli.
• No evidence of aversion was noted when the animals were returned to the restraint box.

## 1 Introduction

There is a tension between some religious requirements and modern commercial stunning methods. For beef to be acceptable to consumers of Halal and Kosher meat, the animal must be alive at the point of exsanguination, undamaged and, if rendered unconscious prior to slaughter, able to recover from the unconscious state if not exsanguinated (Chao, 2022; Farouk et al., 2015; Fuseini et al., 2017; Nakyinsige et al., 2013; Regenstein et al., 2003; Riaz et al., 2021). Commercial stunning methods are used to render the animal unconscious before the slaughtering cut is performed, so that the animal cannot feel any pain associated with the cut, and to improve operator safety, as the unconscious animal does not struggle during the cut. Mechanical stunning (penetrative or non-penetrative) causes damage to the skull and brain tissues; while electrical stunning can result in visible ecchymoses, or blood splashes in the carcase tissues. A dielectric (electromagnetic) system, trademarked DTS: Diathermic Syncope® (DTS) has the potential to address these requirements, without current disadvantages of existing stunning methods. The system, as installed in the Australian facility used in this research, applies electromagnetic energy, at frequency 915 MHz to the forehead of cattle that are restrained in box fitted with a chin lift. DTS application on conscious animals has resulted in rapid onset of unconsciousness, based on the loss of reflexes associated with consciousness and confirmed by electroencephalogram (EEG) (McLean et al., 2017; Rault et al., 2014; Small et al., 2019). Previous research has also shown that the animal has the potential to recover from this unconsciousness, based on return of the corneal and palpebral reflexes, and return of the ability of the eye to focus and follow movement (eye tracking). Further development of the technology indicates that energy deliveries of 150–200 kJ at 18 kW result in minimal physical changes in the brain, and thus the animals would be expected to recover fully. This paper reports on a demonstration of recovery of consciousness post-DTS application in seven cattle.

## 2 Materials and methods

### 2.1 Animal ethics

This study was conducted under the authority of the CSIRO ‘Wildlife and Large Animal’ Animal Ethics Committee, reference 2022–12, in accordance with the provisions of the Australian Code for the care and use of animals for scientific purposes (National Health & Medical Research Council, 2013). Animal numbers were strictly limited by the conditions of the approval.

### 2.2 Method

#### 2.2.1 Study population

Seven *Bos taurus* cattle, designated R1 – R7, liveweight range 470–560 kg, aged 12–16 months, were used for the study (4 heifers; 3 steers). Breeds represented were Australian Angus, Murray Grey and Limousin.

#### 2.2.2 Study design

The study was an observational study conducted over two days in July 2022.

#### 2.2.3 Animal care and handling

Animals were sourced at random from the normal commercial intake at the abattoir and had been held first at pasture at the abattoir for 1 week, then in lairage pens overnight prior to processing. A group of 8 familiar conspecifics were also held in the lairage in an adjacent pen to limit effects of isolation stress.

**Fig. 1:**
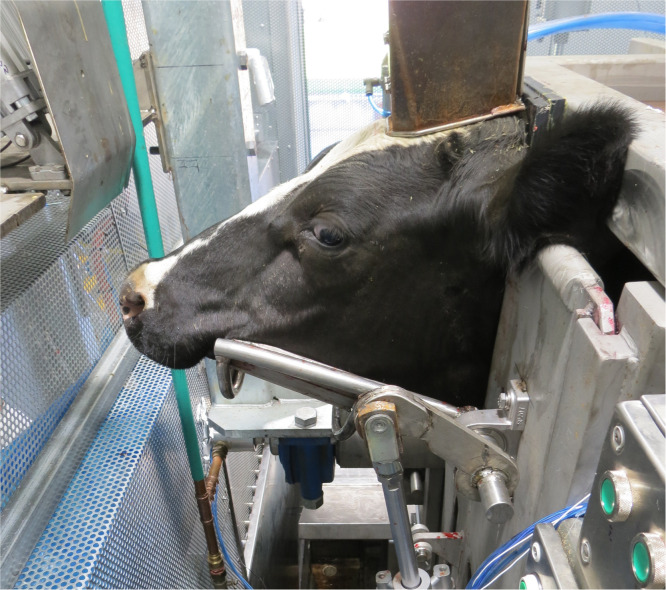
Animal’s head captured in the chin-lift, with waveguide in place, immediately prior to DTS energy application.

Each animal was individually brought to the restraint unit, by an experienced handler using low stress handling techniques (voice and flapper), and the head captured in a chin-lift device (Fig. 1).

#### 2.2.4 Electroencephalography

EEG data were collected as described by Small et al. (2019) from the first six animals prior to and for 3 to 5 min beginning 12 s after the start of DTS application. Briefly, a four-electrode montage was prepared (reference electrode midline over the nasal bones, midway between the nostrils and the eyes; inverting electrodes on the right and left frontal bones, between the eye and the poll; and the ground electrode behind the ear on the bony protruberance of the atlas), using low impedance (< 5 kΩ) electrode pads (RedDotMini, 3 M Australia, North Ryde, NSW) affixed to the skin using cyanoacrylate (Loctite 454, Loctite Australia, Caringbah, NSW). EEG data were collected using an Octal Biolab amplifier and PowerLab (ADInstruments, Sydney, Australia), applying a low-pass filter of 30 Hz. EEG was not recorded from animal 7, such that the handling of this animal prior to DTS application more closely reflected the normal commercial situation. In order to assess the electrical interference associated with energy application, a recording was made during pre-processing testing, whereby a plastic cannister of water was placed under the waveguide while energy was applied. The EEG leads were placed on a shelf next to the cannister so they could record any electrical activity in the environment.

#### 2.2.5 Energy application

DTS energy applications were 180 kJ for animals R1, R2 and R6; 160 kJ for animals R3, R4 and R7; and 150 kJ for animal R5. All applications were at a power level of 18 kW: i.e., the duration of energy application was approximately 10 s for animals R1, R2 and R6, approximately 9 s for animals R3, R4 and R7; and approximately 8 s for animal R5 (kW x time = kJ). The DTS control system measures the energy delivered and energy reflected in real time during application and the time of application is a function of power (kW) and reflected energy. Animal R1 received an interrupted stun: due to operator error (a waveguide restraining strap had not been secured), the waveguide slipped to one side, and the automatic safety cut-out switches were triggered after 20 kJ had been delivered. The waveguide was repositioned, the restraining strap secured, and a further 160 kJ were delivered to meet the 180 kJ target.

#### 2.2.6 Behavioural observations and post-energy-application procedures

The head of the animal was video recorded throughout DTS application, using high-definition closed-circuit television cameras (Dahua HCVR4108HS-S3/8, Zhejiang Dahua Technology Co. Ltd., China). After DTS application, the corneal reflexes, eye position and eye tracking (the ability of the eye to follow movement of a hand passed across the field of vision, approximately 10 cm distance from the eye) were determined to confirm insensibility, and the animal was allowed to return to sensibility while remaining in the restraint unit. When the animal demonstrated awareness of its surroundings (assessed by eye tracking), the EEG leads were removed, the head and neck support were released, and the animal allowed to stand quietly in the restraint unit for a few minutes. Animals one and two appeared very drowsy after recovery of eye reflexes and eye tracking, and refused to back out of the restraint box, so DTS was reapplied, and the carcass processed without delay. Animals three to seven were reversed out of the restraint unit, down the ramp (a distance of 12 m) and placed in a holding pen with a companion animal with hay and water available. The animal entered this holding pen between 2 and 7 min after DTS application. The animal was observed directly for 5 min while it explored its surroundings and settled in the holding pen. Behaviour of the animal in this holding pen was recorded by video (GoPro Hero5, San Mateo, CA, USA) for a period of 20–30 min, then the animal was returned to the restraint unit using low-stress handling techniques, DTS was re-applied, and the carcass was processed. All activities pertaining to one animal were completed and the carcass removed from the bleed rail before the subsequent animal was brought to the restraint unit.

The video footage was watched continuously offline by a single trained observer with over 5 years' experience in livestock behaviour and humane slaughter assessment, to record animal behaviour according to Table 1 and any adverse events. The observer recorded time of commencement and time of cessation of the sub-parameters listed in Table 1, such that duration of individual phases and latencies from onset of energy application could be calculated. The main phases of animal behaviour post energy application are similar to those observed in head-only electrical stunning, namely a tonic or stiff phase followed by a clonic or kicking phase, and then return of brainstem reflexes and ultimately righting reflex and return to full awareness (Beausoleil et al., 2024). As DTS is a novel method for inducing unconsciousness, and precise characterisation of each phase has not yet been published, details of sub parameters that could relate to these phases were included in the ethogram. The design of the rotary box restraint unit fully encloses the animal’s body and precludes accurate assessment of loss of posture or loss of rhythmic breathing based on thoracic movements. Thus, assessment of breathing was conducted based on detection of inspiratory movements at the nostrils.

**Table 1:** Behavioural animal-based measures recorded from video footage during and after energy application, and analysis parameters derived. Ethogram adapted from: EFSA (2004a); Velarde et al. (2002); Verhoeven et al. (2015) and Beausoleil et al. (2024).

Parameter	Sub-parameter	Description
*Tonic convulsive phase*		*Rigid posture of the body with flexion of front and hind legs and/or extension of front legs and neck and flexion of hind legs*
	Tonic Facial muscles	Tensing of muscles around eyes, nose, upper jaw; eyelids held wide open or shut tight, cessation of rhythmic breathing movements at the corner of the nostril.
*Clonic convulsive phase*		*Uncontrolled jerking activity, involuntary running or kicking movements of hind legs and extension with some paddling of front legs. May occur in several phases following tonic convulsive phase*
	Eyelid flutter	Uncontrolled rapid movement of eyelids, rapid blinking
	Ear flicking	Uncontrolled rapid back and forth movements of the ears
	Nose twitching	Uncontrolled rapid movement of the soft end part of the nose adjacent to the nostrils
	Convulsive body/limb movement	Full body jerking motions and/or uncontrolled paddling of the limbs; kicking or walking action
*Return of brainstem reflexes*		*Resumption of normal function of brainstem reflexes such as corneal reflex, palpebral reflex, somatic withdrawal (response to pinching of the nostrils)*
	Rhythmic breathing	Regular movements of the corner of the nostril. Time of return of rhythmic breathing was noted when three evenly-spaced inspirations in series were observed at the nostrils.
	Eyelid blink	Animal opens/closes eyelid on its own without stimulation
	Corneal reflex	Presence of corneal reflex response: retraction of the eyeball, or closure of the eyelids elicited by touching or tapping the cornea with a finger
	Awareness of surroundings	Eye tracks movement passed in front of the eyeball, ears swivel towards sound
	Alertness	Animal lifts and moves head, focuses on personnel activity
*Resumption of postural control*		*The animal attempts to regain posture,* e.g. *purposefully lifts the head out of the yoke. Only movements observed after the end of clonic convulsive activity were considered purposeful and were scored*
	Standing attempt	Movement of limbs after clonic convulsions have ceased.
	Standing unsupported	Hind limbs in a position to support the weight of the hind quarter

#### 2.2.7 EEG data analysis

EEG data were analysed offline using LabChart 8 (ADInstruments, Sydney, Australia). Root mean Square (RMS) Power was calculated from the untransformed, filtered recording. The Spectral Analysis Package within LabChart 8 was used to apply Fast Fourier Transformation (FFT), with multiplication using a Hann window in 1-second epochs with a 25 % overlap. Total power (Ptot) in the entire frequency range (0–30 Hz), median frequency (SEF50) and 95 % Spectral Edge frequency (SEF95) were extracted. Power in each frequency band: Alpha (20.1–30 Hz); Beta (8.1–20 Hz); Theta (4.1–8 Hz) and Delta (0.1–4 Hz) was also extracted. For each animal the median value of Ptot prior to energy application was calculated and this was used as the baseline value. Baseline normalization was then carried out by transforming data for each 1-s epoch into decibel change from baseline according to the formula: dB=10*log10(value/ baseline), to bring all data sets into a comparable format. This process was repeated for each frequency band within each animal. These data, RMS data and data for SEF95 and SEF50 were charted and inspected for EEG suppression and epileptiform activity, and where possible time to resolution of EEG suppression was recorded.

## 3 Results

### 3.1 Behavioural observations

The initial physical response to energy application, which occurred within 1–6 s of the start of energy application (with the exception of animal R1for which energy application was interrupted: it’s tonic response occurred at 10 s), was a tonic phase: sudden tensing of all muscles of the body, with eyes open wide. This was rapidly followed by a period of involuntary movements of the eyelids, ears and muzzle, jerking of the body and convulsive movements of the limb. This convulsive phase began between 0 and 12 s after the start of energy delivery and lasted for 43 – 119 s (Table 2). Convulsive movements were not continuous, rather occurring in bursts of activity, with the movement of facial muscles notably diminishing in intensity towards the end of the convulsive phase. Two animals (R1 and R4) opened their mouths wide at the end of the convulsive phase.

**Table 2:** Expression of behaviours post application of energy, for each animal and arithmetic mean. Latency: time of onset of behaviour after start of energy application (s); Duration: period of time (s) during which the behaviour was observed.

	Animal R1 180 kJ		Animal R2 180 kJ		Animal R3 160 kJ		Animal R4 160 kJ		Animal R5 150 kJ		Animal R6 180 kJ		Animal R7 160 kJ		Mean (*n* = 7)	
	Latency (s)	Duration (s)	Latency (s)	Duration (s)	Latency (s)	Duration (s)	Latency (s)	Duration (s)	Latency (s)	Duration (s)	Latency (s)	Duration (s)	Latency (s)	Duration (s)	Latency (s)	Duration (s)
*Tonic convulsive phase*																
Tonic muscles	10	20	6	10	6	11	1	8	0	13	2	6	4	8	4	11
*Clonic convulsive phase*																
Eyelid flutter	20	57	10	35	13	49	8	22	9	64	6	53	8	51	11	47
Ear flicking	12	73	10	35	13	52	8	24	12	62	6	53	5	54	10	50
Nose twitching	20	81	10	17	13	49	7	39	9	66	6	53	5	54	10	51
Convulsive body/ limb movement	12	115	9	85	7	93	3	43	0	119	2	57	5	54	5	71

The return of rhythmic breathing occurred shortly before the end of the convulsive phase. The exact time of return was difficult to pinpoint, as involuntary movements of the chest muscles occurred as part of the clonic convulsive phase. Time of return of rhythmic breathing was noted when three evenly-spaced breaths were performed in series, which was observed between 45 and 85 s after the start of energy delivery (Table 3). Corneal and palpebral reflexes subsequently returned, with a slow ‘lazy’ response first observed as early as 63 s after the start of energy application, and a crisper response developing as the animal returned to alertness. The exact time of return of the crisp response was difficult to quantify as it depended on the operator checking for a response. Awareness appeared to fluctuate – some animals appeared to regain awareness as early as 83 s after the start of energy application, then lapsed once more into an unresponsive or drowsy state. Full alertness and an interest in the surroundings was present between 119 and 273 s after the start of energy application. There was a failure of video capture for animal R5, due to a loss of power to the camera system, and data for returning reflexes could not be generated.

**Table 3:** Return of behavioural measures associated with consciousness, for each animal and arithmetic mean. Latency: time of return of behaviour after start of energy application (s).

	Animal R1 180 kJ	Animal R2 180 kJ	Animal R3 160 kJ	Animal R4 160 kJ	Animal R5 150 kJ	Animal R6 180 kJ	Animal R7 160 kJ	Mean (*n* = 6)
	Latency (s)	Latency (s)	Latency (s)	Latency (s)	Latency (s)	Latency (s)	q	Latency (s)
*Return of brainstem reflexes*								
Rhythmic breathing	63	85	65	45	Not recorded	59	66	64 (*n* = 6)
Eyelid blink	136	216	216	63	Not recorded	68	71	128 (*n* = 6)
Corneal reflex	Not recorded	218	216	73	Not recorded	90	119	143 (*n* = 5)
Awareness of surroundings	178	228	205	123	Not recorded	90	83	151 (*n* = 6)
Alertness	Not recorded	258	257	273	Not recorded	179	119	217 (*n* = 5)
*Resumption of postural control*								
Standing attempt	189	120	344	366	Not recorded	179	94	215 (*n* = 6)
Standing unsupported	212	459	344	366	Not recorded	315	112	301 (*n* = 6)

Animals R1 and R2 were not removed to the holding pen, as they refused to back up, despite responding to personnel waving a hand in front of the face. The animals remained standing in the restraint, would take one step back, then return to a resting state, shaking their head when tapped by hand on the forehead. After at least 5 min of ineffective encouragement to back up, both were re-stunned, bled and processed for human consumption. The remaining five animals did back up after a few minutes and were returned to the holding pen; all reversed easily out of the restraint unit and down the ramp. Once in the holding pen, which was shared with a familiar conspecific, they explored their surroundings, sniffed at or ate some hay, and then rested (Supplementary video file 1). Drowsiness and mild ataxia were observed when animals were left quiet in the pen; when human activity resumed in the laneway the animals responded in the same manner as the conspecific in the pen: turning towards the noise, ears pricked, and watchful. When herded towards the gate and into the race at the end of the 30-minute observation period, no evidence of ataxia was present and all animals were easily returned to the restraint unit without baulking, backing up or vocalisation. There were no abnormal findings in the carcase or offal.

### 3.2 Electroencephalography

**Fig. 2:**
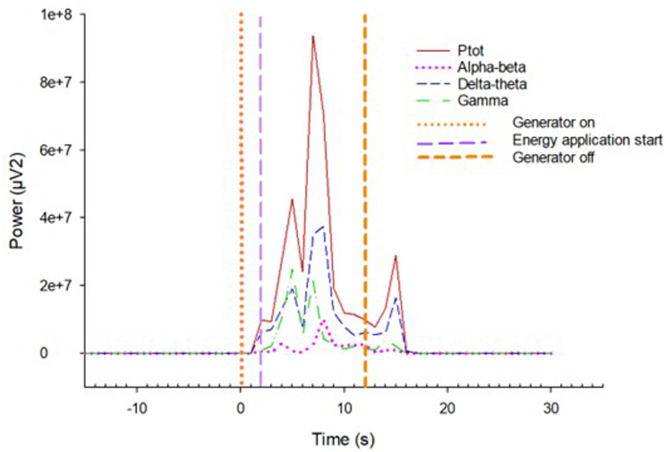
Background electrical interference associated with operation of the generator.

During energy application, EEG cannot be recorded as there is a large amount of electrical interference associated with operation of the generator, obliterating recordings from 1 s prior to energy application until 6 s after the end of energy application (Fig. 2). No electrical interference was detected in association with rolling of the rotary box.

Charts for each individual animals R1 – R6 of: decibel (dB) attenuation in the EEG power spectrum, baseline and 80 s blocks post DTS application; frequency-power distributions, baseline and 30-s blocks post DTS application; RMS power over time; dB change in EEG power over time; dB change in power in frequency bands (Alpha 20.1 - 30 Hz, Beta 8.1 - 20 Hz, Theta 4.1 - 8 Hz and Delta 0.1 - 4 Hz) over time; and change in SEF 95 and SEF 50 over time, are presented in Supplementary File 2.

Root Mean Square (RMS) power in the filtered, but untransformed, signal was initially increased relative to baseline in animals R2, R3, R5 and R6 (Table 4), but decreased in animals R1 and R4. RMS remained suppressed in animal R1 and R4 throughout post-DTS recording (until 282 s post energy application for R1 and until 312 s post energy application for R4). In animals R2 and R3, a, RMS dropped to below baseline after the 42–72 s and 72–102 s intervals, while in animal R5, RMS remained markedly elevated until the 162–192 s interval had elapsed, and less markedly elevated between 192 s and the end of recording at 282 s. RMS in animal R6 was variable throughout recording, with peaks in the 12–42 s, 72–102 s and 132–192 s intervals and RMS below baseline in the 102–132 s and 222–252 s intervals, recording terminated after 252 s.

**Table 4:** Root Mean Square (RMS) power in the untransformed EEG, in a 60 s period prior to energy application (baseline) and 30 s blocks of time post energy application. Post energy application, EEG was recorded from 12 s after the onset of energy delivery. NR: Not recorded.

Time post onset of energy application (s)	Animal	R1	R2	R3	R4	R5	R6
	Energy delivered	180 kJ	180 kJ	160 kJ	160 kJ	150 kJ	180 kJ
0		2.57	11.45	3.33	12.28	1.07	8.16
12–42		0.87	25.51	25.28	7.88	17.51	31.50
42–72		0.59	17.49	16.46	2.29	14.34	8.11
72–102		0.58	7.09	8.25	1.23	13.81	20.89
102–132		0.61	4.92	1.05	0.92	10.31	1.91
132–162		0.73	1.13	2.36	3.52	19.17	10.95
162–192		0.73	1.96	2.16	0.72	17.39	17.99
192–222		0.84	6.94	3.72	2.54	5.24	7.63
222–252		0.62	5.67	NR	2.51	6.50	1.15
252–282		0.63	20.18	NR	1.96	2.55	NR
282–312		NR	NR	NR	1.15	NR	NR

**Fig. 3:**
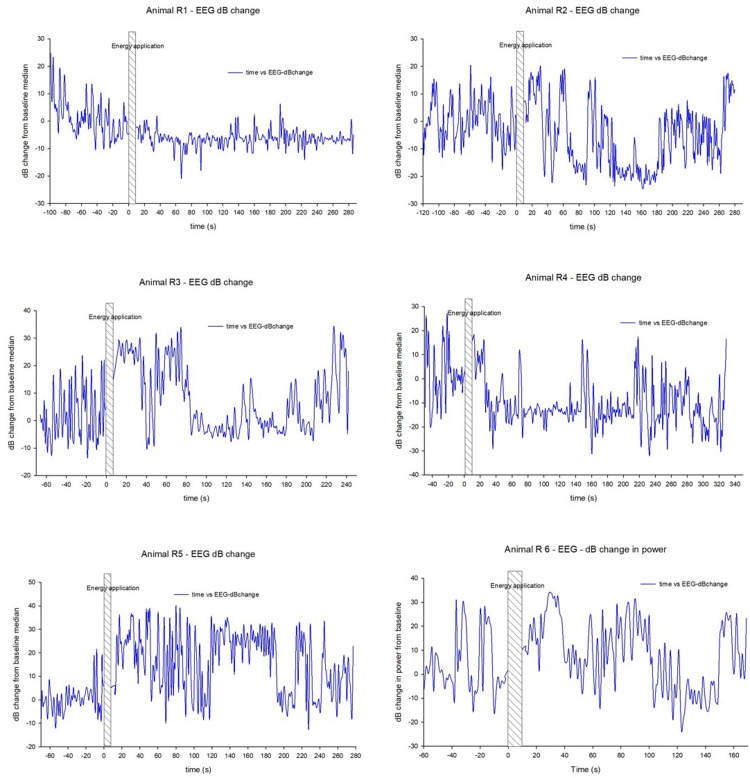
Decibel change in EEG power over time for each animal. Energy application is represented by the hatched bar in each chart.

Decibel change in EEG power (Fig. 3) mirrored the changes in RMS. In animal R1, there was a sustained reduction in power relative to baseline, animal R2 showed initial increase in power, reducing over the first 60 s to a minimum, with a short period of increase between 90 and 120 s, then again suppressed until 180 s. Animal R3 showed an increase relative to baseline for the first 80 s, then reduction till 201 s, with the occasional spikes around 140–150 s. Animal R4 showed an increase in the initial 30 s post DTS application, followed by suppression till 210 s, with the occasional spike at 70 s, and 150–160 s. Animal R5 showed an increase relative to baseline, while animal R6 showed an initial increase for the first 100 s, followed by suppression. dB change in power in each of the frequency bands (Alpha 20.1 - 30 Hz, Beta 8.1 - 20 Hz, Theta 4.1 - 8 Hz and Delta 0.1 - 4 Hz) followed the same pattern as overall power (Supplementary File 2).

**Fig. 4:**
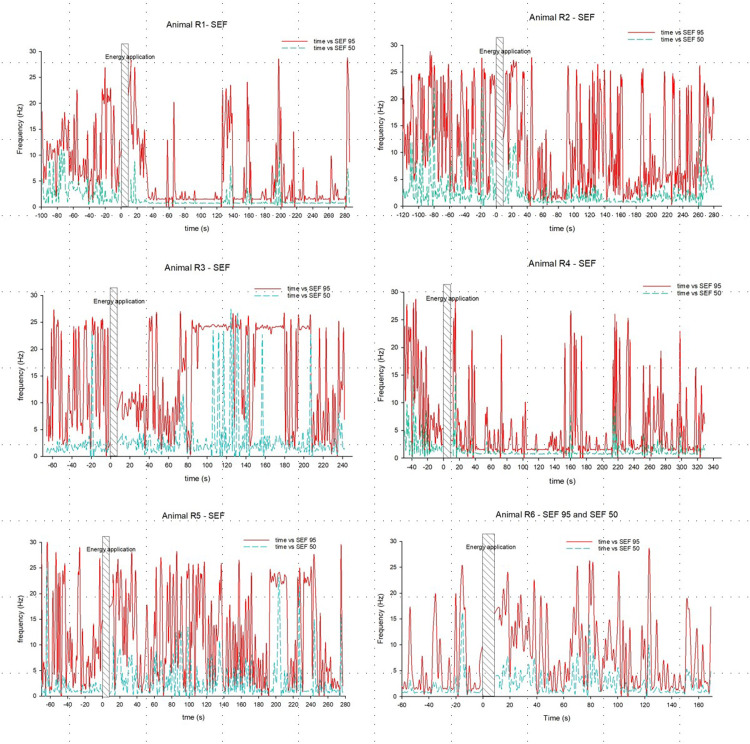
SEF 95 and SEF 50 over time for each animal. Energy application is represented by the hatched bar in each chart.

Following DTS application, there was a shift in frequency:power distribution in each 30 s block, with reductions in the higher frequencies in the EEG relative to lower frequencies, particularly after the initial 42 s (Supplementary File 2). The second-by-second assessments (Fig. 4) showed reductions in SEF 95 from 30 s post DTS application, with spikes at 55–70 s, 125–140 s, 155–165 and intermittently from 190 s in Animal R1, and reductions in SEF 50 from 20 s, with spikes at 130–140 s, 155–165 s and 190–200 s. Animal R2 showed reductions in SEF 95 between 40 and 90 s, with some spiking, and again from 160–260 s, while SEF 50 was reduced from 30–90 s and 140–180 s post DTS application. Animal R3 showed a drop in SEF 95 from DTS application to 70 s, with some spikes at 40–50 s; the impact on SEF 50 was less evident. Both SEF 95 and SEF 50 were reduced, with intermittent spiking. In animal R4 from 40 to 150 s post DTS application, and again from 175–210 s. The impact of SEF 95 and SEF 50 in animal R5 were not clear, while in animal R6, there appeared to be an increase in SEF 95 and SEF 50 post DTS application.

## 4 Discussion

As described in Small et al. (2019), who reported on earlier prototypes of the microwave stunning system, induction of unconsciousness was characterized by onset of a tonic-clonic convulsive state, which was maintained for at least 43 s after the start of energy application. In the current study, the initial tonic state occurred between 0 and 10 s after the start of energy application, and full clonic convulsions were evident thereafter. These clonic convulsions occurred in intermittent bursts of activity, as described for smallstock undergoing electrical stunning (Beausoleil et al., 2024).

The exact mechanism by which unconsciousness is induced and then maintained is not completely understood. The presence of an initial tonic-clonic response aligns with the responses of cattle, calves, sheep and goats to electrical stunning (Beausoleil et al., 2024; EFSA, 2004b; Von Holleben et al., 2010). However the duration of the tonic-clonic state in the current study is more consistent and longer than that reported for electrical stunning of cattle by Wotton et al. (2000), who describe an initial tonic phase of 1–23 s and a clonic phase of 1–87 s, while the overall duration of unconsciousness is greater that described for electrical stunning (EFSA, 2004b). For a pulsed ultra-high current electrical stun, the description of the behavioural response aligns with that observed in the current study: Robins et al. (2014) described a ‘tonic or shuddering’ phase, which aligns with a tonic-clonic response, lasting for 5.3–14.1 s, followed by a ‘stationary’ phase, with eye reflexes returning after an average of 31 s (minimum interval not reported). In contrast, in the current study, eye reflexes returned at the earliest, 80 s after the start of energy application. It is likely that the extended interval between induction of unconsciousness and return of eye reflexes is a result of thermal inactivation of neurotransmission within the brain. Thermal unconsciousness, such as that induced by exercise heat stress or fever, is reported to occur when core body temperatures reach between 40 and 45 °C (Hjeresen et al., 1983; Lerman et al., 2014; McDaniel et al., 1991; Mohanty et al., 1997; Ohshima et al., 1992; Roccatto et al., 2010; Yoshizawa et al., 2016). Under hyperthermic conditions, the extensive circulatory network of the brain assists in maintaining brain temperatures 1–2 °C below core body temperature (Hjeresen et al., 1983), suggesting that thermal unconsciousness is achieved when the brain temperature reaches between 39–44 °C. In rats, at a brain temperature of 41 °C (5.4 °C above control brain temperature of 35.6 °C), adenosine triphosphate (ATP) concentration was reduced by 29.2 % and creatine phosphate (CP) concentration was reduced by 44 % (Sanders & Joines, 1984). In the same study, nicotinamide adenine dinucleotide (NADH) fluorescence increased during microwave application and returned to baseline levels within one minute after cessation of energy application. Another study (Ikarashi et al., 1984) demonstrated that there was a rapid reduction in acetylcholinesterase activity in rat brains once a temperature of 50 °C was reached.

During energy application, at the beginning of the tonic-clonic phase, there was a temporary cessation of breathing. The exact time of return of rhythmic breathing was difficult to pinpoint, as involuntary movements of the chest muscles occurred as part of the clonic convulsive phase. Time of return of rhythmic breathing was noted when three evenly-spaced breaths were performed in series, which was observed between 45 and 85 s after the start of energy delivery. As described in pigs recovering from electrical stunning, the return of rhythmic breathing coincides with the end of the clonic convulsive phase (Anil, 1991). The character of breathing after DTS was slow and deep, suggesting that the respiratory centres are affected but not obliterated. As in chemically induced general anaesthesia, loss of reflexes (and subsequent return of reflexes) occurs in a specific order, related to the depth of anaesthesia, and also related to the order in which cranial nerves leave the brainstem. The cranial nerves, in descending order are: I Olfactory nerve; II Optic nerve; III Oculomotor nerve; IV Trochlear nerve; V Trigeminal nerve; VI Abducent nerve; VII Facial nerve; VIII Vestibulocochlear nerve; IX Glossopharyngeal nerve; X Vagus nerve; XI Spinal division of accessory nerve; XII Hypoglossal nerve. Thus, under general anaesthesia, the earliest signs of returning consciousness, are voluntary tongue movements (Cranial nerve XII) and swallowing or gag reflex (Cranial nerves IX and X); while loss of spontaneous breathing is an early indicator that anaesthetic depth is too great. Cranial nerve X also modulates cardiac and respiratory rate – lesions in cranial nerve X can result in tachycardia and hyperventilation, while stimulation of cranial nerve X can result in bradycardia and deeper/slower breathing (King, 1987). Following the return of the gag reflex, the next sense to return is hearing (cranial nerve VIII), which is challenging to assess in an abattoir environment. Following this, reflexes associated with the eye return. Cranial nerves III to VII control the muscular responses to stimuli detected by cranial nerves I and II, but until cranial nerves I and II return to function, our ability to assess function of cranial nerves III to VII is limited. Cranial nerve II is the afferent, or sensory nerve associated with the eye responses: pupillary reflex, menace reflex, corneal reflex and fixating response (ability to focus and follow movement with the eyes). Thus, as soon as corneal response is confirmed, full consciousness may be close (Anil, 1991; Terlouw et al., 2016). However, with DTS, there appears to be a lag phase during which a slow, or ‘lazy’ reflex can be elicited from the eye, while the pupil remains dilated and the eye staring. This slow response can also be observed in electrical stunning (M.H. Anil, personal communication). Furthermore, a corneal reflex can be observed in some cases of electrical stunning, in the absence of other signs of consciousness (Blackmore & Newhook, 1982; Blackmore & Petersen, 1981; Vogel et al., 2011). When the response to a touch on the eyeball becomes crisp and rapid, full alertness is imminent. Returning reflexes and early signs of eye tracking and awareness could be detected as early as 63 s after the start of energy application, but animals appeared drowsy and lethargic for the first 2–3 min, after which they could be backed down the lead-in race and returned to a holding pen. The two animals that would not back down the race were both Australian Angus animals and had been observed to be very quiet animals while in lairage prior to the study day. After recovery, they would stand quietly in the restraint box and resist encouragement to walk backwards. They would take a step or two backwards, but then stop, look around and walk forward again into the restraint box. Rags, flappers and coats were waved in front of them, but they still refused to walk back, rather becoming obstinate and headbutting the rag or coat. Our Animal Ethics Authority has a maximum time limit for maintaining the animals after recovery, and when the animals still had not walked back after 15–20 min after the start of energy application, we made the call to re-stun and process the animals. This did affect the number of animals observed in the holding pen, but does not affect the main conclusion, namely that animals can recover fully from the unconsciousness induced.

The electroencephalogram (EEG) has long been considered to be the gold standard method of assessing consciousness or lack thereof, however, in both electrical stunning and in the current study, the period during and immediately after application, the EEG recording is saturated and heavily contaminated by artefact, firstly associated with the energy application itself, and then with the muscular twitches of the tonic-clonic phase (Mason et al., 2017; Robins et al., 2014). When the body can be held reasonably still, as in the current study, reductions in F50 and suppression of Ptot can be identified, sustained for a duration of at least 60–90 s, after which the amplitude of the EEG began to increase, but as with the studies by Robins et al. (2014) and Beausoleil et al. (2024), had not returned to baseline levels by the end of the recording period.

The results here contrast somewhat with earlier publications of the DTS system, which suggested that there was a sustained grand-mal epileptiform state evident on EEG when higher energy levels were applied (Rault et al., 2014; Small et al., 2019). Notwithstanding the refinements to the delivery apparatus implemented after generation of those data, the energy applied to the animals participating in the current study is in the range 150 – 180 kJ, at a power of 18 kW, whereas the energy applications described in Rault et al. (2014) and Small et al. (2019) were in the ranges of 200 – 360 kJ, at power levels of 20 – 30 kW. A reduced level of electrical excitation in the brain cells would be expected in the current study as compared to those previous studies.

The current study clearly demonstrates that cattle rendered unconscious using the DTS system at 18 kW, 160–180 kJ, can fully regain consciousness and behave in a normal manner when returned to a pen with a conspecific. The duration of the observation period was limited to 30 min by the conditions of ethical approval, but there were no indications that the animal could not have continued to function normally. An important observation was that animals were easily returned to the restraint unit without baulking, suggesting that the DTS treatment was either not aversive, or the animals had no memory of a negative experience in the box. This lack of aversiveness aligns with the findings of Leach et al. (1980), who used conditioned stimuli to assess the experience of sheep undergoing reversible electric stunning. After 11 daily applications of stunning, conditioned to be associated with a bright light, exposure to the light alone resulted in no physiological (heart rate, plasma glucose and packed cell volume) responses in the sheep (*n* = 20), leading the authors to conclude that electrical stunning was not aversive.

One of the concerns raised by religious authorities around stunning prior to slaughter is that the animals should be alive at the time of slaughter, such that if at the last moment prior to the neck cut, if it was deemed that the animal were not needed for meat production, it should be able to be returned to the herd or flock (Fuseini et al., 2017; Riaz et al., 2021; Sazili et al., 2023). In the UK, the Agriculture and Horticulture Development Board (AHDB) operate a “Demonstration of Life Protocol” for sheep stunned by head-only electrical stunning, to allow validation of recoverability (https://projectblue.blob.core.windows.net/media/Default/Trade/Halal/Factsheet.pdf). Under this assurance protocol, abattoirs may apply, and gain approval, to conduct a maximum of two demonstrations per year, in which two animals (sheep or goats only) may be held after electrical stunning for a maximum of 60 s without exsanguination, while observations for return of rhythmic breathing are conducted. When two rhythmic breaths have occurred, the animals must be re-stunned and slaughtered. It is clear that cattle undergoing DTS application at 150–180 kJ will comply with these requirements and may even be able to return to herd life.

## 5 Limitations

Animal numbers and methodology used were limited by the Animal Ethics Approval. The short post-recovery observation period was insufficient to allow generation of a time budget, and a formal assessment against a defined ethogram, or a control group of animals, was not performed.

The methodology used in this study to record EEG used non-invasive electrodes under the conditions of the animal ethics approval, and poor contact between the electrode pad and the skin may have affected data collection. A key limitation in the evaluation of the DTS system is the inability to assess EEG during induction of unconsciousness. Although electrocorticography (ECoG) may reduce the artefact associated with muscular twitches, the recording will still be saturated during energy application. Furthermore, although a 30 Hz low-pass filter was applied to the EEG data, electromyelographic interference may have been present, particularly during the tonic-clonic phase.

## 6 Conclusions

Using energy applications of between 150 and 180 kJ energy at 18 kW ensures loss of consciousness within 10 s of the start of energy application, of a duration of 63 s or more (based on returning palpebral or corneal reflexes), with a long transition to full return of consciousness.

Animals could recover fully from the induced unconsciousness and responded normally to environmental stimuli. No evidence of aversion was noted when these animals were returned to the restraint box a second time, some 30 min after recovery.

## Animal ethics

This study was conducted under the authority of the CSIRO ‘Wildlife and Large Animal’ Animal Ethics Committee, reference 2022–12, in accordance with the provisions of the Australian Code for the care and use of animals for scientific purposes (National Health & Medical Research Council, 2013). Animal numbers were strictly limited by the conditions of the approval.

## Funding

This research was funded by Wagstaff Food Services Pty Ltd. Contract number 2022,040,702.

## CRediT authorship contribution statement

**Alison Small:** Writing – original draft, Visualization, Validation, Supervision, Project administration, Methodology, Investigation, Funding acquisition, Formal analysis, Data curation, Conceptualization. **Ian Jenson:** Writing – original draft, Visualization, Validation, Formal analysis. **Andrew Phillips:** Resources, Project administration, Methodology, Investigation, Conceptualization. **David McLean:** Visualization, Validation, Supervision, Software, Resources, Methodology, Investigation, Data curation, Conceptualization. **Troy Kalinowski:** Resources, Methodology, Investigation, Data curation. **James Ralph:** Validation, Supervision, Resources, Methodology, Investigation, Funding acquisition, Conceptualization.

## Declaration of competing interest

James Ralph is the commercialiser of the DTS: Diathermic Syncope® technology, funding provider and facilitator of the research. All data collection, data curation, analytical activities and interpretation were performed by the remaining authors, who declare that these activities were conducted in the absence of any commercial or financial relationships that could be construed as a potential conflict of interest.

## Appendix Supplementary materials

Image, application 1

Image, video 2

## References

[bib0001] Anil M.H. (1991). Studies on the return of physical reflexes in pigs following electrical stunning. Meat Science.

[bib0002] Beausoleil N.J., Farouk M.M., Webster J., Johnson C.B., Dowling S., Sazili A.Q., Cameron C. (2024). Comparison of recovery of sheep, goats, and calves from reversible electrical head-only and head-to-body stunning for halal meat production. New Zealand Veterinary Journal.

[bib0003] Blackmore D.K., Newhook J.C. (1982). Electroencephalographic studies of stunning and slaughter of sheep and calves - part 3: The duration of insensibility induced by electrical stunning in sheep and calves. Meat Science.

[bib0004] Blackmore D.K., Petersen G.V. (1981). Stunning and slaughter of sheep and calves in New Zealand. New Zealand Veterinary Journal.

[bib0005] Chao E.-C. (2022). Islam and veterinary science: rethinking animal suffering through Islamic animal ethics and the evolving definition of halal slaughter [Original Research]. Frontiers in Veterinary Science.

[bib0006] EFSA (2004). Opinion of the scientific panel on animal health and welfare on a request from the commission related to welfare aspects of the main systems of stunning and killing the main commercial species of animals. EFSA Journal.

[bib0007] EFSA (2004). Welfare aspects of animal stunning and killing methods. Scientific Panel for Animal Health and Welfare.

[bib0008] Farouk M.M., Regenstein J.M., Pirie M.R., Najm R., Bekhit A.E.D., Knowles S.O. (2015). Spiritual aspects of meat and nutritional security: Perspectives and responsibilities of the Abrahamic faiths. Food Research International.

[bib0009] Fuseini A., Wotton S.B., Hadley P.J., Knowles T.G. (2017). The compatibility of modern slaughter techniques with halal slaughter: A review of the aspects of 'modern' slaughter methods that divide scholarly opinion within the Muslim community. Animal Welfare.

[bib0010] Hjeresen D.L., Guy A.W., Petracca F.M., Diaz J. (1983). A microwave-hyperthermia model of febrile convulsions. Bioelectromagnetics.

[bib0011] Ikarashi Y., Maruyama Y., Stavinoha W.B. (1984). Study of the use of the microwave magnetic-field for the rapid inactivation of brain-enzymes. Japanese Journal of Pharmacology.

[bib0012] King A.S. (1987).

[bib0013] Leach T.M., Warrington R., Wotton S.B. (1980). Use of a conditioned-stimulus to study whether the initiation of electrical pre-slaughter stunning is painful. Meat Science.

[bib0014] Lerman O., Bruchim Y., Kelmer E., Lenchner I. (2014). Concurrent heatstroke and insulinoma in a dog: A case report. Israel Journal of Veterinary Medicine.

[bib0015] Mason A., Tolo E., Haga H.A., Ieee (2017). 11th International Conference on Sensing Technology (ICST).

[bib0016] McDaniel H.B., Jenkins R.L., McDaniel H.G. (1991). Experimental hyperthermia - protective effect of oxygen carrying fluorocarbon and crystalloids intraperitoneally. American Journal of the Medical Sciences.

[bib0017] McLean D., Meers L., Ralph J., Owen J.S., Small A. (2017). Development of a microwave energy delivery system for reversible stunning of cattle. Research in Veterinary Science.

[bib0018] Mohanty D., Gomez J., Mustafa K.Y., Khogali M., Das K.C. (1997). Pathophysiology of bleeding in heat stress: An experimental study in sheep. Experimental Hematology.

[bib0019] Nakyinsige K., Che Man Y.B., Aghwan Z.A., Zulkifli I., Goh Y.M., Abu Bakar F., Al-Kahtani H.A., Sazili A.Q. (2013). Stunning and animal welfare from Islamic and scientific perspectives. Meat Science.

[bib0020] National Health and Medical Research Council (2013). Australian code for the care and use of animals for scientific purposes. National Health and Medical Research Council.

[bib0021] Ohshima T., Maeda H., Takayasu T., Fujioka Y., Nakaya T. (1992). An autopsy case of infant death due to heat-stroke. American Journal of Forensic Medicine and Pathology.

[bib0022] Rault J.L., Hemsworth P.H., Cakebread P.L., Mellor D.J., Johnson C.B. (2014). Evaluation of microwave energy as a humane stunning technique based on electroencephalography (EEG) of anaesthetised cattle. Animal Welfare.

[bib0023] Regenstein J.M., Chaudry M.M., Regenstein C.E. (2003). The kosher and halal food laws. Comprehensive Reviews in Food Science and Food Safety.

[bib0024] Riaz M.N., Irshad F., Riaz N.M., Regenstein J.M. (2021). Pros and cons of different stunning methods from a Halal perspective: A review. Translational Animal Science.

[bib0025] Robins A., Pleiter H., Latter M., Phillips C.J.C. (2014). The efficacy of pulsed ultrahigh current for the stunning of cattle prior to slaughter. Meat Science.

[bib0026] Roccatto L., Modenese A., Occhionero V., Barbieri A., Serra D., Miani E., Gobba F. (2010). Heat stroke in the workplace: Description of a case with fatal outcome. Medicina Del Lavoro.

[bib0027] Sanders A.P., Joines W.T. (1984). The effects of hyperthermia and hyperthermia plus microwaves on rat-brain energy-metabolism. Bioelectromagnetics.

[bib0028] Sazili A.Q., Kumar P., Hayat M.N. (2023). Stunning compliance in Halal slaughter: A review of current scientific knowledge. Animals.

[bib0029] Small A., Lea J., Niemeyer D., Hughes J., McLean D., Ralph J. (2019). Development of a microwave stunning system for cattle 2: Preliminary observations on behavioural responses and EEG. Research in Veterinary Science.

[bib0030] Terlouw C., Bourguet C., Deiss V. (2016). Consciousness, unconsciousness and death in the context of slaughter. Part II. Evaluation methods. Meat Science.

[bib0031] Velarde A., Ruiz-de-la-Torre J.L., Rosello C., Fabrega E., Diestre A., Manteca X. (2002). Assessment of return to consciousness after electrical stunning in lambs. Animal Welfare.

[bib0032] Verhoeven M.T.W., Gerritzen M.A., Kluivers-Poodt M., Hellebrekers L.J., Kemp B. (2015). Validation of behavioural indicators used to assess unconsciousness in sheep. Research in Veterinary Science.

[bib0033] Vogel K.D., Badtram G., Claus J.R., Grandin T., Turpin S., Weyker R.E., Voogd E. (2011). Head-only followed by cardiac arrest electrical stunning is an effective alternative to head-only electrical stunning in pigs. Journal of Animal Science.

[bib0034] Von Holleben, K., Von Wenzlawowicz, M., Gregory, N., Anil, H., Velarde, A., Rodriguez, P., Cenci-Goga, B., Catanese, B., & Lambooij, B. (2010). Report on good and adverse practices - animal welfare concerns in relation to slaughter practices from the viewpoint of veterinary sciences. *1.3*, 81. Retrieved 1/11/2021, from https://www.dialrel.net/dialrel/images/veterinary-concerns.pdf.

[bib0035] Wotton S.B., Gregory N.G., Whittington P.E., Parkman I.D. (2000). Electrical stunning of cattle. Veterinary Record.

[bib0036] Yoshizawa T., Omori K., Takeuchi I., Miyoshi Y., Kido H., Takahashi E., Jitsuiki K., Ishikawa K., Ohsaka H., Sugita M., Yanagawa Y. (2016). Heat stroke with bimodal rhabdomyolysis: A case report and review of the literature. Journal of Intensive Care.

